# Characterization of “*Candidatus* Ehrlichia Pampeana” in *Haemaphysalis juxtakochi* Ticks and Gray Brocket Deer (*Mazama gouazoubira*) from Uruguay

**DOI:** 10.3390/microorganisms9102165

**Published:** 2021-10-17

**Authors:** María Laura Félix, Sebastián Muñoz-Leal, Luis Andrés Carvalho, Diego Queirolo, Susana Remesar, María Teresa Armúa-Fernández, José Manuel Venzal

**Affiliations:** 1 Laboratorio de Vectores y Enfermedades Transmitidas, Departamento de Ciencias Biológicas, CENUR Litoral Norte—Salto, Universidad de la República, Rivera 1350, Salto 50000, Uruguay; m.teresa.armua@gmail.com (M.T.A.-F.); dpvuru@hotmail.com (J.M.V.); 2 Departamento de Ciencia Animal, Facultad de Ciencias Veterinarias, Universidad de Concepción, Av. Vicente Méndez 595, Casilla 537, Chillán 3780000, Chile; sebamunoz@udec.cl; 3 AgResearch, Grasslands Research Centre, Private Bag 11008, Palmerston North 4442, New Zealand; luisandrescarvalho@gmail.com; 4 Laboratorio de Ecología de Vertebrados, CENUR Noreste, Universidad de la República, Ituzaingó 667, Rivera 40000, Uruguay; dqueirolo@cur.edu.uy; 5 INVESAGA Group, Department of Animal Pathology, Faculty of Veterinary Sciences, Universidade de Santiago de Compostela, 27002 Lugo, Spain; susana.remesar@usc.es

**Keywords:** Rickettsiales, Anaplasmataceae, *Ehrlichia*, molecular characterization, ticks, *Haemaphysalis juxtakochi*, gray brocket deer, Uruguay

## Abstract

Human ehrlichiosis are scantily documented in Uruguay. The aim of this study was to investigate the presence of *Ehrlichia* spp. in *Haemaphysalis juxtakochi* and in a gray brocket deer (*Mazama gouazoubira*) from Uruguay. The presence of *Ehrlichia* DNA was investigated in free-living *H. juxtakochi* in five localities of southeast and northeast Uruguay, as well as blood, spleen, and ticks retrieved from a *M. gouazoubira*. *Ehrlichia* spp. DNA was detected in six out of 99 tick pools from vegetation, in the spleen of *M. gouazoubira,* and in one out of five pools of ticks feeding on this cervid. Bayesian inference analyses for three loci (*16S* rRNA, *dsb,* and *groEL*) revealed the presence of a new rickettsial organism, named herein as “*Candidatus* Ehrlichia pampeana”. This new detected *Ehrlichia* is phylogenetically related to those found in ticks from Asia, as well as *Ehrlichia ewingii* from USA and Cameroon. Although the potential pathogenicity of “*Ca*. E. pampeana” for humans is currently unknown, some eco-epidemiological factors may be relevant to its possible pathogenic role, namely: (i) the phylogenetic closeness with the zoonotic agent *E. ewingii*, (ii) the evidence of *H. juxtakochi* parasitizing humans, and (iii) the importance of cervids as reservoirs for zoonotic *Ehrlichia* spp. The molecular detection of “*Ca.* E. pampeana” represents the third *Ehrlichia* genotype described in Uruguay.

## 1. Introduction

Ehrlichiae are small Gram-negative tick-transmitted coccobacilli that obligately dwell inside cells. These microorganisms are classified as α-proteobacteria belonging to the family Anaplasmataceae included in the order Rickettsiales [1]. Wild mammals, and probably birds [2], constitute natural vertebrate hosts for *Ehrlichia* spp., which are horizontally transmitted through tick bites [3]. *Ehrlichia* species infect different cells in mammals and ticks. While monocytes, neutrophils, or endothelial cells have been detected as the mammalian target cells, salivary glands, intestinal epithelium, and hemolymph cells, are infected in the vectors [4]. Some *Ehrlichia* spp. exhibit tropism for mononuclear phagocyttablees and compose microcolonies within membrane-bound cytoplasmic vacuoles known as morulae [5]. These bacteria are the agents of ehrlichiosis, a complex of life-threatening emerging zoonoses and diseases of worldwide veterinary concern [6]. The genus *Ehrlichia* currently consists of six validly published species, namely *Ehrlichia canis*, *Ehrlichia chaffeensis*, *Ehrlichia ewingii*, *Ehrlichia minasensis*, *Ehrlichia muris*, and *Ehrlichia ruminantium* [7]. For all these species, sequences of the complete chromosome are already available in genetic databases [3]. Four species (*E. chaffeensis*, *E. ewingii*, *E. canis*, and *E. muris*) are known to infect humans and cause potentially severe to fatal ehrlichiosis [7]. Cervids (Cervidae) have been demonstrated to be reservoirs of human pathogenic Ehrlichiae, particularly *E. chaffeensis*, *E. ewingii,* and *E. muris* [8,9,10,11,12].

Recent molecular studies performed in South America have unveiled novel genotypes of *Ehrlichia* retrieved from domestic and wild vertebrates, as well as from their ticks [2,13]. For instance, in Uruguay there is only one characterization of *Ehrlichia* which corresponds to two novel genotypes currently found in *Ixodes auritulus* [14]. There are several studies reporting the evidence of deer as reservoirs of pathogenic *Ehrlichia* spp. in northern latitudes of the globe [9,10]. Despite the report of a case of autochthonous human ehrlichiosis in 2001 [15], this disease in Uruguay has unclear status. The fact that *I. auritulus* does not bite humans [16] prompted us to consider the presence of other tick vectors of *Ehrlichia* spp., and that the disease could be underdiagnosed in this country. Interestingly, *Haemaphysalis juxtakochi* is one of the five species of ticks that have been reported parasitizing humans in Uruguay [16], and cervids are common hosts mainly for its adult stages [17,18]. Therefore, the present study aimed to investigate the presence of *Ehrlichia* spp. in *H. juxtakochi* and its host, the gray brocket deer (*Mazama gouazoubira*) in Uruguay.

## 2. Materials and Methods

Between March 2014 and August 2017 field work was conducted in five localities of Uruguay: Gruta de los Cuervos (31°37′08″ S, 56°02′47″ W), Tacuarembó Department; Amarillo (31°39′49″ S, 55°03′02″ W) and Lunarejo (31°08′29″ S, 55°54′01″ W), Rivera Department; Reserva Natural Salus (34°25′16″ S, 55°18′54″ W), Lavalleja Department; and Laguna Negra (34°05′09″ S, 53°44′17″ W), Rocha Department.

Ticks were collected from vegetation using the flagging method as described previously [14] and stored in plastic containers with 95% ethanol. In addition, a juvenile female *M. gouazoubira* carcass killed by dogs in September 2017 at Gruta de los Cuervos, Tacuarembó Department was included in this study. Ticks and a sample from the spleen and blood were retrieved from the carcass and stored at −20 °C until use. Ticks were identified using a Nikon stereo microscope SMZ1000 following morphological keys for larval, nymph, and adult stages [17,19]. Since *Ehrlichia* species are not maintained by transovarial transmission [20], only nymphs and adult ticks were analyzed in this study. Ticks were pooled according to sex, developmental stage, site, collection date, and host. Ticks were rinsed with distilled water to remove ethanol, and the ticks were cut thoroughly with dissecting scissors. DNA was extracted using a GeneJET Genomic DNA Purification kit (Thermo Scientific, Vilnius, Lithuania), according to manufacturer’s instructions. DNA concentration was estimated using a Nanodrop 2000 spectrophotometer (Thermo Scientific, Wilmington, DE, USA).

For *Ehrlichia* DNA detection, a molecular screening targeting a fragment of the *16S* rRNA gene of the Anaplasmataceae family was carried out using primers and protocols as described previously [21]. Subsequently, positive samples were subjected to two additional PCR protocols to amplify a nearly full-length sequence (~1431 bp) of the *16S* rRNA gene based on two overlapping fragments [22,23]. In addition, a nested and a heminested PCR targeting partial fragments of *groEL* (60 kDa chaperonin) and *dsb* (disulfide oxireductase) genes, respectively, were performed [24,25,26,27,28]. All primers used in this study and fragment sizes are listed in Table 1. Distilled water and DNA of *E. canis* were included as negative and positive controls, respectively. PCR products were analyzed by electrophoresis in 1.5% agarose gels. Amplicons were purified using a GeneJET PCR purification kit (Thermo Fisher Scientific, Vilnius, Lithuania) and sent for sequencing to Macrogen (Seoul, Korea). BLASTn analyses (www.ncbi.nlm.nih.gov/blast, accessed date: 4 October 2021) were performed in order to infer closest identities with microorganisms available in the GenBank database [29], and to include those sequences in a phylogenetic analysis.

Phylogenies for the genus *Ehrlichia* were constructed with sequences of each amplified gene and GenBank-retrieved homologues. The alignments for *16S* rRNA, *dsb,* and *groEL* were implemented in CLUSTAL W [30]. We used Bayesian inferences to reconstruct evolutionary relationships in the genus with MrBayes 3.2.5 [31]. The general time reversible (GTR) model was selected to run all the phylogenies using 1,000,000 generations. Each tree was sampled every 100 generations, beginning with random seeds, and ran four times. The first 25% of the trees were considered burn-in, and the remaining trees used to calculate Bayesian posterior probabilities. Sequences of *Neoehrlichia mikurensis* (EU810406; AB213021) and *E. ruminantium* (AF308669) rooted the phylogenetic trees.

## 3. Results

A total of 5772 *H. juxtakochi* ticks (89 females, 107 males, 1681 nymphs, and 3895 larvae) were collected from vegetation in nineteen samplings carried out in the five selected localities (Table S1). Additionally, 18 *H. juxtakochi* (five females, four males, and nine nymphs) were obtained from the of *M. gouazoubira* carcass.

For *Ehrlichia* DNA detection, 1864 *H. juxtakochi* collected from vegetation (85 females, 98 males, and 1681 nymphs) were divided in 99 samples and analyzed (Table 2). The ticks were processed individually or grouped in pools containing 2 to 62 specimens collected upon the vegetation. The samples examined from *M. gouazoubira* were: five pools containing *H. juxtakochi* ticks (one containing five females, one with four males, and three with three nymphs each) as well as a blood and a spleen sample.

Six out of the 99 *H. juxtakochi* samples containing ticks from the vegetation were positive (6.1%) (4 out of 71 nymph pools, 1 out of 14 male pools, and 1 of 14 female pools) (Table 2). Partial sequences generated for *16S* rRNA, *dsb,* and *groEL* loci of *Ehrlichia* sp. were deposited in GenBank with the accession numbers listed in Table 2. Moreover, one out of the three nymph pools containing *H. juxtakochi* specimens retrieved from *M. gouazoubira* was positive (GenBank accession numbers: MZ733621, MZ779087, and MZ779096 for *16S* rRNA, *dsb,* and *groEL*, respectively). For the samples of blood and spleen of *M. gouazoubira* only a partial sequence for *groEL* of *Ehrlichia* sp. was obtained from the spleen (GenBank accession number MZ779099).

The comparison among the sequences obtained herein revealed a similarity percentage of 100 and between 99.38–100% and 99.69–100% for *16S* rRNA, *groEL,* and *dsb* fragment genes, respectively. Analyses of the *16S* rRNA sequences retrieved from *H. juxtakochi* (610 to 1234 bp) revealed 100% identity with *Ehrlichia* sp. clone HLAE331 obtained from *Haemaphysalis longicornis* from South Korea (GenBank accession number: GU075697) and 99.92% with two sequences named as *Ehrlichia* sp. TC249-2 and *Ehrlichia* sp. TC251-2 from *Dermacentor nuttalli* from China (KJ410252-KJ410253). Sequences of the *16S* rRNA gene of other *Ehrlichia* spp. obtained from ticks from different parts of the world were <90% identical. Accordingly, partial sequences of *groEL* obtained from *H. juxtakochi* and spleen of *M. gouazoubira* (1140 and 1242 bp, respectively) also showed high identity (97.73–97.91%) with sequences from the *Ehrlichia* sp. detected in *D. nuttalli* from China (KJ410294-KJ410296). In contrast, the closest identity for the *dsb* gene (295 to 330 bp) was *E. ewingii,* detected in *Amblyomma americanum* from USA (AY428950: 90.25%) and from a dog from Cameroon (DQ151999: 90.10%).

**Figure 1:**
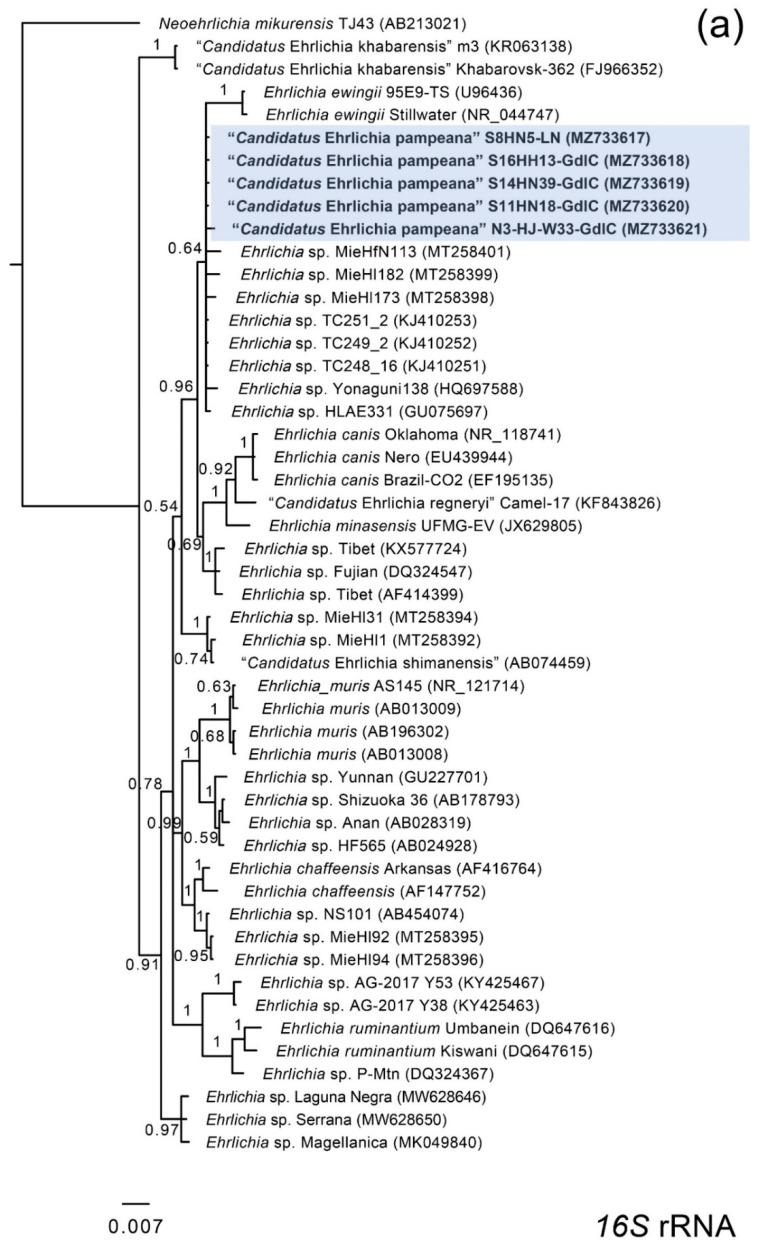

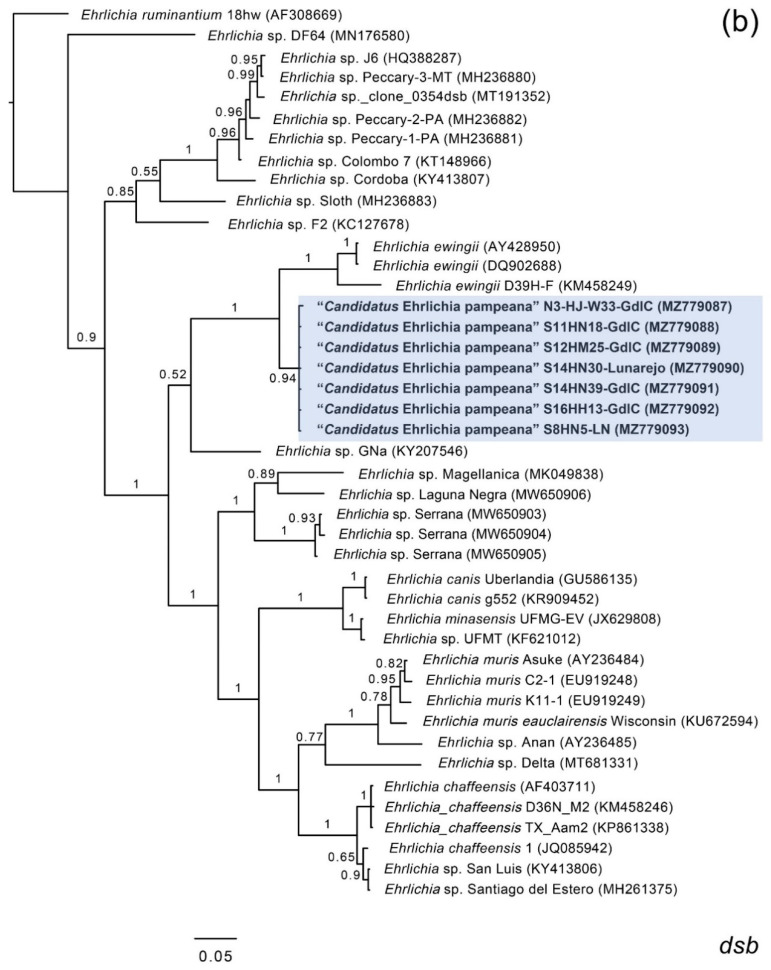

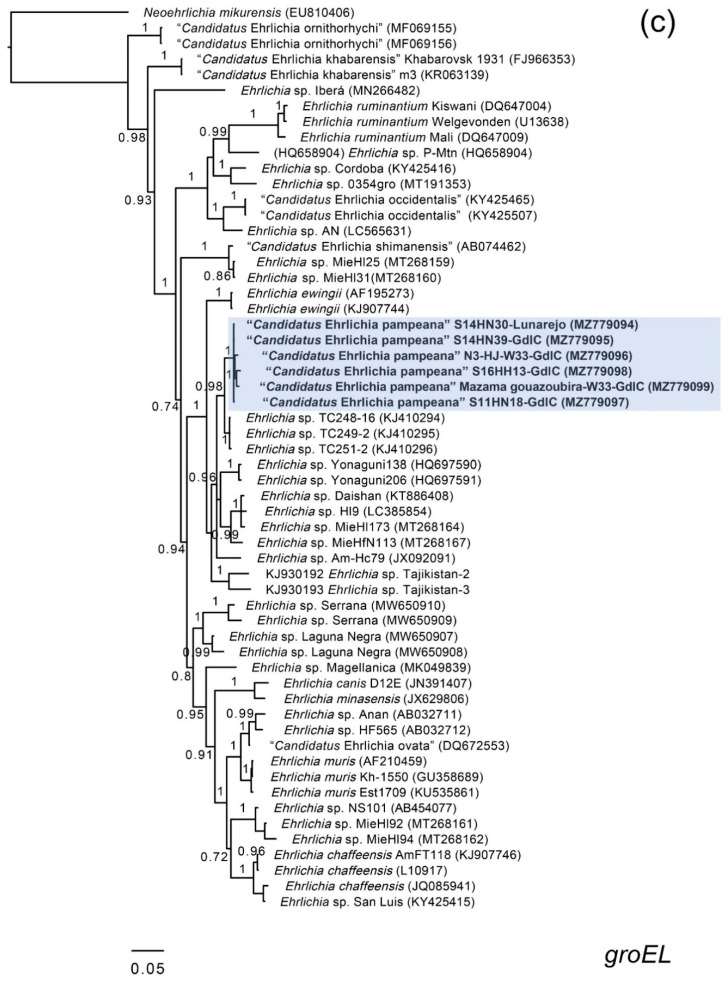
Bayesian phylogenetic analyses inferred for partial fragments of the genes (**a**) *16S* rRNA, (**b**) *dsb,* and (**c**) *groEL*. Bayesian posterior probabilities are indicated upon each branch. The positions of “*Candidatus* Ehrlichia pampeana” are highlighted with blue. Scale bar indicates the number of substitutions per nucleotide position. GenBank accession numbers are in brackets.

The phylogenetic relationship of characterized *Ehrlichia* genes was assessed through Bayesian analyses. Phylogenetic trees constructed with partial sequences of *16S* rRNA and *groEL* produced similar topologies (Figure 1a,c). Although with low support, the *Ehrlichia 16S* rRNA sequences obtained in this study formed a clade with sequences of *Ehrlichia* spp. characterized from *H. longicornis* (HQ697588, MT258398, MT258399) and *Haemaphysalis flava* (MT258401) from Japan, and *D. nuttalli* from China (KJ410251–KJ410253) (Figure 1a). Similarly to the results obtained for the *16S* rRNA gene, the *groEL* sequences formed a clade with sequences of *Ehrlichia* spp. from *Haemaphysalis*, *Dermacentor,* and *Hyalomma* ticks from Asia, as well as *E. ewingii* from a human and *A. americanum* from USA (AF195273, KJ907744) (Figure 1c). In contrast, *dsb* sequences clustered with *E. ewingii* homologues retrieved from *A. americanum* and *Amblyomma inornatum* from USA (AY428950, KM458249), and *Rhipicephalus sanguineus* from Cameroon (DQ902688) (Figure 1b).

## 4. Discussion

In recent decades, molecular advances have favored the determination of novel species and strains of *Ehrlichia* in ticks from South America; for instance, *E. minasensis* and *E. canis* in *Rhipicephalus microplus* and *R. sanguineus,* respectively [32]. In addition, *Ehrlichia* cf. *chaffeensis*, and a series of strains (*Ehrlichia* sp. strain Córdoba, *Ehrlichia* sp. strain San Luis, *Ehrlichia* sp. strain Iberá, *Ehrlichia* sp. strain Jaguar, *Ehrlichia* sp. strain Delta, *Ehrlichia* sp. strain La Dormida, and a *Ehrlichia* sp.) were detected in ticks of the genus *Amblyomma* [13,33,34,35,36,37,38,39]. Recently, new *Ehrlichia* genotypes were described in *Ixodes uriae* from Chile and *Ixodes auritulus* from Uruguay [2,14]. Collectively, these findings suggest that different *Ehrlichia* species and genotypes are circulating in South American ecosystems.

The genetic distances and phylogenetic relationships for the *16S* rRNA, *groEL,* and *dsb* genes of the *Ehrlichia* sp. characterized in this study clearly denote the finding of a novel species related to the *Ehrlichia* species harbored by *Haemaphysalis* spp., *Hyalomma anatolicum,* and *D. nuttalli* ticks from Asia [3,40,41,42,43,44,45]. We propose its denomination as “*Candidatus* Ehrlichia pampeana”. The species name is in allusion to the Pampa biome where positive ticks and deer were found. Remarkably, “*Ca.* E. pampeana” is also related to *E. ewingii* detected in *Amblyomma* spp. and dog blood from USA and *R. sanguineus* from Cameroon [46,47,48,49,50]. The topology of the phylogenetic trees also suggested that “*Ca.* E. pampeana” is closely related to *E. ewingii*. Although *16S* rRNA and *groEL* phylogenies indicated that other *Ehrlichia* spp. detected in ticks from Asia clustered with “*Ca.* E. pampeana”, there are no *dsb* sequences available for the Asian Ehrlichiae genotypes, thus no phylogenetic relationship could be established with this locus (Figure 1b).

“*Candidatus* E. pampeana” is associated with the gray brocket deer since a fragment of the *groEL* gene was retrieved from the spleen of this cervid. This *Ehrlichia* sp. deer association was previously reported for two human-pathogenic *Ehrlichia* species such as *E. chaffeensis* and *E. ewingii* that use *Odocoileus virginianus* (white-tailed deer) as their main animal reservoir in the USA [9]. This fact, added to the detection of “*Ca.* E. pampeana” DNA in *H. juxtakochi* ticks, suggests that *M. gouazoubira* could act as a reservoir for this bacterium, which could be transmitted by its associated tick species (*H. juxtakochi).*

The molecular characterization of “*Ca.* E. pampeana” represents the third genotype of *Ehrlichia* determined in Uruguay, and the first report of an *Ehrlichia* sp. in *H. juxtakochi*, as well as for *Haemaphysalis* spp. from South America.

Human ehrlichiosis was documented in Uruguay more than ten years ago [15], and further cases have not been reported. Notably, *Amblyomma triste*, a tick that commonly bites humans in South America, has been positive to *Ehrlichia* spp. detection in Brazil and Argentina [37,51]; however, bacteria of this genus have not been detected in *A. triste* Uruguayan populations to date [37,52]. While the pathogenicity of “*Ca*. E. pampeana” for humans is uncertain, our results highlight eco-epidemiological features that might be relevant to suggest this novel *Ehrlichia* as a putative human pathogen as follows: (i) “*Ca*. E. pampeana” is phylogenetically closely related to *E. ewingii*, a recognized zoonotic pathogen [7], (ii) although *H. juxtakochi* parasitizes cervids, it has been also recorded feeding on humans [16,17], and (iii) the role of cervids as reservoirs for pathogenic *Ehrlichia* species has been previously suggested in North America [9].

Ehrlichiae are transstadially transmitted bacteria that need vertebrate hosts in order to thrive in nature [1]. For this reason, more studies are needed to determine the presence of “*Ca*. E. pampeana” in different *H. juxtakochi* hosts along the distribution range of this tick species. Moreover, further studies will be necessary to understand the eco-epidemiology of this novel bacteria and to assess its pathogenicity.

## Figures and Tables

**Table 1:** PCR primers used to amplify the partial *16S* rRNA, *groEL*, and *dsb* genes of *Ehrlichia* spp.

Primer Name	Targeted Gene	Sequence (5′–3′)	Amplicon Size (bp)	Reference
EHR16SD * EHR16SR * fD1	*16S* rRNA	GGT ACC YAC AGA AGA AGT CC	345	[21]
		TAG CAC TCA TCG TTT ACA GC		[21]
		AGA GTT TGA TCC TGG CTC AG	~1431	[22,23]
rP2		ACG GCT ACC TTG TTA CGA CTT		[22,23]
HS1a	*groEL*	AIT GGG CTG GTA ITG AAA T	~1400	[24,26]
HS6a		CCI CCI GGI ACI AIA CCT TC		[24,26]
HS43		ATW GCW AAR GAA GCA TAG TC	1297	[25]
HSVR		CTC AAC AGC AGC TCT AGT AGC		[25]
Dsb-330	*dsb*	GAT GAT GTT TGA AGA TAT SAA ACA AAT	401	[27,28]
Dsb-720 ** Dsb-380		CTA TTT TAC TTC TTA AAG TTG ATA WAT C	349	[27,28]
		ATT TTT AGR GAT TTT CCA ATA CTT GG		[28]

* Primers used in the initial PCR screening, ** primer used on first and second round.

**Table 2:** Data of *Haemaphysalis juxtakochi* collected in vegetation for each site and processed for detection of “*Candidatus* Ehrlichia pampeana”.

Collection Site	Stage	N° of Ticks Processed	Pools	Positive Pools	Positive Pools Code	GenBank Accession Numbers		
						*16S* rRNA	*dsb*	*groEL*
Gruta de los Cuervos (T)	Female	65	7	1	S16HH13	MZ733618	MZ779092	MZ779098
	Male	77	8	1	S12HM25	-	MZ779089	-
	Nymph	969	29	2	S11HN18 S14HN39	MZ733620 MZ733619	MZ779088 MZ779091	MZ779097 MZ779095
Amarillo (Ri)	Female	0	0	0				
	Male	0	0	0				
	Nymph	12	2	0				
Lunarejo (Ri)	Female	10	3	0				
	Male	15	4	0				
	Nymph	401	15	1	S14HN30	-	MZ779090	MZ779094
Reserva Natural Salus (L)	Female	3	2	0				
	Male	4	1	0				
	Nymph	124	7	0				
Laguna Negra (Ro)	Female	7	2	0				
	Male	2	1	0				
	Nymph	175	18	1	S8HN5	MZ733617	MZ779093	-
Total		1864	99	6				

(T) Tacuarembó, (Ri) Rivera, (L) Lavalleja, (Ro) Rocha.

## Data Availability

All data presented in this study are available in the article.

## Supplementary Materials

The following are available online at https://www.mdpi.com/article/10.3390/microorganisms9102165/s1, Table S1: Complete data of *Haemaphysalis juxtakochi* collected in vegetation for each site and detection of “*Candidatus* Ehrlichia pampeana”.

## References

[1] Ismail N., Bloch K.C., McBride J.W. (2010). Human ehrlichiosis and anaplasmosis. Clin. Lab. Med..

[2] Muñoz-Leal S., Clemes Y.S., Lopes M.G., Acosta I.C.L., Serpa M.C.A., Mayorga L.F.S.P., Gennari S.M., González-Acuña D., Labruna M.B. (2019). Novel *Ehrlichia* sp. detected in Magellanic penguins (*Sphenicus magellanicus*) and in the seabird tick *Ixodes uriae* from Magdalena Island, southern Chile. Ticks Tick Borne Dis..

[3] Su H., Onoda E., Tai H., Fujita H., Sakabe S., Azuma K., Akachi S., Oishi S., Abe F., Ando S. (2021). Diversity unearthed by the estimated molecular phylogeny and ecologically quantitative characteristics of uncultured *Ehrlichia* bacteria in *Haemaphysalis* ticks, Japan. Sci. Rep..

[4] Brouqui P., Matsumoto K., Raoult D., Parola P. (2007). Bacteriology and phylogeny of Anaplasmataceae. Rickettsial Diseases.

[5] Paddock C.D., Childs J.E. (2003). *Ehrlichia chaffeensis*: A prototypical emerging pathogen. Clin. Microbiol. Rev..

[6] Esemu S.N., Ndip L.M., Ndip R.N. (2011). *Ehrlichia* species, probable emerging human pathogens in sub-Saharan Africa: Environmental exacerbation. Rev. Environ. Health.

[7] Lin M., Xiong Q., Chung M., Daugherty S.C., Nagaraj S., Sengamalay N., Ott S., Godinez A., Tallon L.J., Sadzewicz L. (2021). Comparative analysis of genome of *Ehrlichia* sp. HF, a model bacterium to study fatal human Ehrlichiosis. BMC Genom..

[8] Yabsley M.J., Varela A.S., Tate C.M., Dugan V.G., Stallknecht D.E., Little S.E., Davidson W.R. (2002). *Ehrlichia ewingii* infection in white-tailed deer (*Odocoileus virginianus*). Emerg. Infect. Dis..

[9] Paddock C.D., Yabsley M.J. (2007). Ecological havoc, the rise of white-tailed deer, and the emergence of *Amblyomma americanum*-associated zoonoses in the United States. Curr. Top. Microbiol. Immunol..

[10] Tamamoto C., Seino N., Suzuki M., Kaji K., Takahashi H., Inokuma H. (2007). Detection of *Ehrlichia muris* DNA from sika deer (*Cervus nippon yesoensis*) in Hokkaido, Japan. Vet. Parasitol..

[11] Nair A.D.S., Cheng C., Jaworski D.C., Willard L.H., Sanderson M.W., Ganta R.R. (2014). *Ehrlichia chaffeensis* infection in the reservoir host (white-tailed deer) and in an incidental host (dog) is impacted by its prior growth in macrophage and tick cell environments. PLoS ONE.

[12] Allerdice M.E.J., Hecht J.A., Karpathy S.E., Paddock C.D. (2017). Evaluation of Gulf Coast ticks (Acari: Ixodidae) for *Ehrlichia* and *Anaplasma* species. J. Med. Entomol..

[13] Muraro L.S., Nogueira M.F., Borges A.M.C.M., Souza A.O., Vieira T.S.W.J., Aguiar D.M. (2021). Detection of *Ehrlichia* sp. in *Amblyomma sculptum* parasitizing horses from Brazilian wetland. Ticks Tick Borne Dis..

[14] Félix M.L., Muñoz-Leal S., Carvalho L.A., Queirolo D., Remesar Alonso S., Nava S., Armúa-Fernández M.T., Venzal J.M. (2021). Molecular characterization of novel *Ehrlichia* genotypes in *Ixodes auritulus* from Uruguay. CRPVBD.

[15] Conti-Díaz I.A. (2001). Enfermedades emergentes y reemergentes en Uruguay. Rev. Med. Urug..

[16] Guglielmone A., Robbins R. (2018). Hard Ticks (Acari: Ixodida: Ixodidae) Parasitizing Humans. A Global Review.

[17] Nava S., Venzal J.M., González-Acuña D., Martins T.F., Guglielmone A.A. (2017). Ticks of the Southern Cone of America.

[18] Guglielmone A.A., Nava S., Robbins R.G. (2021). Neotropical Hard Ticks (Acari: Ixodida: Ixodidae). A Critical Analysis of Their Taxonomy, Distribution, and Host Relationships.

[19] Kohls G.M. (1960). Records and new synonymy of New World *Haemaphysalis* ticks, with descriptions of the nymph and larva of *H. juxtakochi* Cooley. J. Parasitol..

[20] Ismail N., McBride J.W. (2017). Tick-borne emerging infections: Ehrlichiosis and anaplasmosis. Clin. Lab. Med..

[21] Parola P., Roux V., Camicas J.L., Baradji I., Brouqui P., Raoult D. (2000). Detection of Ehrlichiae in African ticks by polymerase chain reaction. Trans. R. Soc. Trop. Med. Hyg..

[22] Weisburg W.G., Bams S.M., Pelletier D.A., Lane D.J. (1991). 16S Ribosomal DNA amplification for phylogenetic study. J. Bacterial..

[23] Inokuma H., Parola P., Raoult D., Brouqui P. (2001). Molecular survey of *Ehrlichia* infection in ticks from animals in Yamaguchi Prefecture, Japan. Vet. Parasitol..

[24] Sumner J.W., Nicholson W.L., Massung R.F. (1997). PCR Amplification and Comparison of Nucleotide Sequences from the *groESL* heat shock operon of *Ehrlichia* species. J. Clin. Microbiol..

[25] Lotric-Furlan S., Petrovec M., Zupanc T.A., Nicholson W.L., Sumner J.W., Childs J.E., Strle F. (1998). Human Granulocytic Ehrlichiosis in Europe: Clinical and laboratory findings for four patients from Slovenia. Clin. Infect. Dis..

[26] Nicholson W.L., Castro M.B., Kramer V.L., Sumner J.W., Childs J.E. (1999). Dusky-footed wood rats (*Neotoma fuscipes*) as reservoirs of granulocytic ehrlichiae (Rickettsiales: Ehrlichieae) in northern California. J. Clin. Microbiol..

[27] Doyle C.K., Labruna M.B., Breitschwerdt E.B., Tang Y., Corstvet R.E., Hegarty B.C., Bloch K.C., Li P., Walker D.H., McBride J.W. (2005). Detection of medically important *Ehrlichia* by quantitative multicolor TaqMan real-time PCR of the *dsb* Gene. J. Mol. Diagn..

[28] Almeida A., Souza T., Marcili A., Labruna M. (2013). Novel *Ehrlichia* and *Hepatozoon* agents infecting the crab-eating fox (*Cerdocyon thous*) in southeastern Brazil. J. Med. Entomol..

[29] Altschul S.F., Gish W., Miller W., Myers E.W., Lipman D.J. (1990). Basic local alignment search tool. J. Mol. Biol..

[30] Thompson J.D., Higgins D., Gibson T.J. (1994). CLUSTALW: Improving the sensitivity of progressive multiple sequence alignment through sequence weighting position-specific gap penalties and weight matrix choice. Nucleic. Acids. Res..

[31] Huelsenbeck J.P., Ronquist F. (2001). MRBAYES: Bayesian inference of phylogenetic trees. Bioinformatics.

[32] Cabezas Cruz A., Zweygarth E., Vancová M., Broniszewska M., Grubhoffer L., Passos L., Ribeiro M.B., Alberdi P., de la Fuente J. (2016). *Ehrlichia minasensis* sp. nov., isolated from the tick *Rhipicephalus microplus*. Int. J. Syst. Evol. Microbiol..

[33] Tomassone L., Nuñez P., Gurtler R., Ceballos L.A., Orozco M.A., Kitron U.D., Farber M. (2008). Molecular detection of *Ehrlichia chaffeensis* in *Amblyomma parvum* ticks, Argentina. Emerg Infect Dis..

[34] Cicuttin G.L., De Salvo M.N., Nava S. (2017). Two novel *Ehrlichia* strains detected in *Amblyomma tigrinum* ticks associated to dogs in peri-urban areas of Argentina. Comp Immunol Microbiol Infect Dis..

[35] Guillemi E.C., Orozco M.M., Argibay H.D., Farber M.D. (2019). Evidence of *Ehrlichia chaffeensis* in Argentina through molecular detection in marsh deer (*Blastocerus*
*dichotomus*). Int. J. Parasitol. Parasites Wildl..

[36] Monje L.D., Fernandez C., Percara A. (2019). Detection of *Ehrlichia* sp. Strain San Luis and ‘*Candidatus* Rickettsia andeanae’ in *Amblyomma parvum* ticks. Ticks Tick Borne Dis..

[37] Cicuttin G.L., De Salvo M.N., Díaz Pérez P., Silva D., Félix M.L., Venzal J.M., Nava S. (2020). A novel *Ehrlichia* strain (Rickettsiales: Anaplasmataceae) detected in *Amblyomma triste* (Acari: Ixodidae), a tick species of public health importance in the Southern Cone of America. Pathog. Glob. Health.

[38] Eberhardt A.T., Fernandez C., Fargnoli L., Beldomenico P.M., Monje L.D. (2020). A putative novel strain of *Ehrlichia* infecting *Amblyomma tigrinum* associated with Pampas fox (*Lycalopex gymnocercus*) in Esteros del Iberá ecoregion, Argentina. Ticks Tick Borne Dis..

[39] Fargnoli L., Fernandez C., Monje L.D. (2020). Novel *Ehrlichia* Strain Infecting Cattle Tick *Amblyomma neumanni*, Argentina, 2018. Emerg Infect Dis..

[40] Oh J.Y., Moon B.C., Bae B.K., Shin E.H., Ko Y.H., Kim Y.J., Park Y.H., Chae J.S. (2009). Genetic identification and phylogenetic analysis of *Anaplasma* and *Ehrlichia* species in *Haemaphysalis longicornis* collected from Jeju Island, Korea. J. Bacteriol. Virol..

[41] Matsumoto K., Takeuchi T., Yokoyama N., Katagiri Y., Ooshiro M., Zakimi S., Gaowa, Kawamori F., Ohashi N., Inokuma H. (2011). Detection of the new *Ehrlichia* species closely related to *Ehrlichia ewingii* from *Haemaphysalis longicornis* in Yonaguni Island, Okinawa, Japan. J. Vet. Med. Sci..

[42] Kang Y.J., Diao X.N., Zhao G.Y., Chen M.H., Xiong Y., Shi M., Fu W.M., Guo Y.J., Pan B., Chen X.P. (2014). Extensive diversity of Rickettsiales bacteria in two species of ticks from China and the evolution of the Rickettsiales. BMC Evol. Biol..

[43] Luo L., Sun J., Yan J., Wang C., Zhang Z., Zhao L., Han H., Tong Z., Liu M., Wu Y. (2016). Detection of a novel *Ehrlichia* species in *Haemaphysalis longicornis* tick from China. Vector Borne Zoonotic Dis..

[44] Taira M., Ando S., Kawabata H., Fujita H., Kadosaka T., Sato H., Monma N., Ohashi N., Saijo M. (2019). Isolation and molecular detection of *Ehrlichia* species from ticks in western, central, and eastern Japan. Ticks Tick Borne Dis..

[45] Kartashov M.Y., Kononova Y.V., Petrova I.D., Tupota N.L., Mikryukova T.P., Ternovoi V.A., Tishkova F.H., Loktev V.B. (2020). Detection of *Ehrlichia* sand *Theileria* sin *Hyalomma anatolicum* ticks collected in Tajikistan. Vavilovskii Zh Genet Sel..

[46] Anderson B.E., Greene C.E., Jones D.C., Dawson J.E. (1992). *Ehrlichia ewingii* sp. nov., the etiologic agent of canine granulocytic ehrlichiosis. Int. J. Syst. Bacteriol..

[47] Goldman E.E., Breitschwerdt E.B., Grindem C.B., Hegarty B.C., Walls J.J., Dumler J.S. (1998). Granulocytic ehrlichiosis in dogs from North Carolina and Virginia. J. Vet. Intern. Med..

[48] Ndip L.M., Ndip R.N., Ndive V.E., Awuh J.A., Walker D.H., McBride J.W. (2007). *Ehrlichia* species in *Rhipicephalus sanguineus* ticks in Cameroon. Vector Borne Zoonotic Dis..

[49] Labruna M.B., McBride J.W., Camargo L.M., Aguiar D.M., Yabsley M.J., Davidson W.R., Stromdahl E.Y., Williamson P.C., Stich R.W., Long S.W. (2007). A preliminary investigation of *Ehrlichia* species in ticks, humans, dogs, and capybaras from Brazil. Vet. Parasitol..

[50] Medlin J.S., Cohen J.I., Beck D.L. (2015). Vector potential and population dynamics for *Amblyomma inornatum*. Ticks Tick-Borne Dis..

[51] Widmer C.E., Azevedo F.C., Almeida A.P., Ferreira F., Labruna M.B. (2011). Tickborne bacteria in free-living jaguars (*Panthera onca*) in Pantanal, Brazil. Vector Borne Zoonotic Dis..

[52] Venzal J.M., Estrada-Peña A., Portillo A., Mangold A.J., Castro O., de Souza C.G., Félix M.L., Pérez-Martínez L., Santibánez S., Oteo J.A. (2008). Detection of alpha and gamma-proteobacteria in *Amblyomma triste* (Acari: Ixodidae) from Uruguay. Exp. Appl. Acarol..

