# Supplementary material for: Characterization of “Candidatus Ehrlichia Pampeana” in Haemaphysalis juxtakochi Ticks and Gray Brocket Deer (Mazama gouazoubira) from Uruguay
Source: Microorganisms. 2021 Oct 17;9(10):2165. doi: 10.3390/microorganisms9102165 (PMC8538733; doi:10.3390/microorganisms9102165)
Supplement: Supplementary file 1 [file microorganisms-09-02165-s001.zip › microorganisms-1386998-supplementary.pdf]

microorganisms

MDPI

Supplementary Materials

Table S1. Complete data of *Haemaphysalis juxtakochi* collected in vegetation for each site and detection of "*Candidatus* Ehrlichia pampeana".

Collection site	Date	Stage	N° ticks	Pools	Positive pools
Gruta de los Cuervos (T)	August 2016	Female	19	2	0
		Male	28	3	0
		Nymph	152	3	1
		Larva	379	-	-
	October 2016	Female	21	1	0
		Male	23	1	1
		Nymph	362	7	0
		Larva	49	-	-
	December 2016	Female	17	2	0
		Male	16	2	0
		Nymph	163	4	1
		Larva	0	-	-
	April 2017	Female	1	1	1
		Male	2	1	0
		Nymph	55	5	0
		Larva	574	-	-
	July 2017	Female	7	1	0
		Male	8	1	0
		Nymph	99	4	0
		Larva	948	-	-
	August 2017	Female	4	0	0
		Male	9	0	0
		Nymph	138	6	0
		Larva	457	-	-
Subtotal			3531	44	4
Amarillo (Ri)	October 2016	Female	0	0	0
		Male	0	0	0
		Nymph	11	1	0
		Larva	0	-	-
	June 2017	Female	0	0	0
		Male	0	0	0
		Nymph	1	1	0
		Larva	2	-	-
Subtotal			14	2	0
Lunarejo (Ri)	October 2016	Female	3	1	0
		Male	4	1	0
		Nymph	122	4	0
		Larva	70	-	-
	December 2016	Female	4	1	0
		Male	5	1	0
		Nymph	110	3	1
		Larva	0	-	-
	April 2017	Female	0	0	0
		Male	1	1	0
		Nymph	27	3	0

		Larva	319	-	-
	July 2017	Female	3	1	0
		Male	5	1	0
		Nymph	142	5	0
		Larva	541	-	-
Subtotal			1356	22	1
Reserva Natural Salus (L)	May 2016	Female	0	0	0
		Male	0	0	0
		Nymph	29	2	0
		Larva	71	-	-
	December 2016	Female	2	1	0
		Male	4	1	0
		Nymph	46	3	0
		Larva	1	-	-
	July 2017	Female	1	1	0
		Male	0	0	0
		Nymph	49	2	0
		Larva	410	-	-
Subtotal			613	10	0
Laguna Negra (Ro)	March 2014	Female	0	0	0
		Male	0	0	0
		Nymph	9	1	0
		Larva	35	-	-
	May 2014	Female	1	1	0
		Male	0	0	0
		Nymph	8	1	0
		Larva	26	-	-
	August 2014	Female	0	0	0
		Male	2	1	0
		Nymph	70	7	0
		Larva	10	-	-
	November 2014	Female	6	1	0
		Male	0	0	0
		Nymph	88	9	1
		Larva	3	-	-
Subtotal			258	21	1
Total			**5772**	**99**	**6**

(T) Tacuarembó, (Ri) Rivera, (L) Lavalleja, (Ro) Rocha.
